# Warming delays the phenological sequences of an autumn‐flowering invader

**DOI:** 10.1002/ece3.4177

**Published:** 2018-05-24

**Authors:** Yang Peng, Jian‐Xia Yang, Xiao‐Hui Zhou, Pei‐Hao Peng, Jing‐Ji Li, Wei‐Ming He

**Affiliations:** ^1^ State Key Laboratory of Vegetation and Environmental Change Institute of Botany Chinese Academy of Sciences Beijing China; ^2^ College of Resources and Environment University of Chinese Academy of Sciences Beijing China; ^3^ Ecological Resources and Landscape Research Institute Chengdu University of Technology Chengdu China; ^4^ College of Environment and Civil Engineering Chengdu University of Technology Chengdu China

**Keywords:** climate warming, invasion stage, nitrogen deposition, phenophase onset, *Solidago canadensis*

## Abstract

Phenology can play an important role in driving plant invasions; however, little is known about how climate warming, nitrogen (N) deposition, and invasion stages influence the phenological sequences of autumn‐flowering invaders in a subtropical climate. Accordingly, we conducted an experiment to address the effects of experimental warming, N‐addition, and community types on the first inflorescence buds, flowering, seed‐setting, and dieback of invasive *Solidago canadensis*. Warming delayed the onset of first inflorescence buds, flowering, seed‐setting, and dieback; N‐addition did not influence these four phenophases; community types influenced the onset of first seed‐setting but not the other phenological phases. Seed‐setting was more sensitive to experimental manipulations than the other phenophases. The onset of first inflorescence buds, flowering, and seed‐setting was marginally or significantly correlated with ramet height but not ramet numbers. Our results suggest that future climate warming might delay the phenological sequences of autumn‐flowering invaders and some phenophases can shift with invasion stages.

## 1 INTRODUCTION

The success of invasive plants can be ascribed to a suite of contributors. Mounting evidence suggests that plant phenology may play a pivotal role in driving plant invasions (Fridley, 2012; Godoy, Castro‐Diez, Valladares, & Costa‐Tenorio, 2009; Novy, Flory, & Hartman, 2013; Rejmánek, 2013; Smith & Hall, 2016; Smith & Reynolds, 2015; Wolkovich & Cleland, 2011). Harrington, Brown, and Reich (1989) pioneered work in this aspect, and their research has brought renewed interest in the study of plant phenology (Cleland, Chuine, Menzel, Mooney, & Schwartz, 2007). Overall, invasive plants occupy unique phenological niches that confer them with growth or competitive advantages over native species (Fridley, 2012; Wolkovich & Cleland, 2011; Wolkovich et al., 2013). For example, invasive plants commonly exhibit extended leaf phenology, which grants them longer intervals between leaf budbreak in spring and abscission in autumn relative to co‐occurring natives and allows them to exploit light and nutrients unavailable to dormant natives (Fridley, 2012; Rejmánek, 2013; Wolkovich & Cleland, 2011). Some invaders also have extended flowering and fruiting phenology (Rejmánek, 2013). Additionally, phenological plasticity of an invader may be related to its invasiveness potential (Godoy et al., 2009).

The need to study phenology in plant invasions has become widely recognized, but several aspects remain to be poorly understood. First, the majority of phenological studies have been conducted in temperate or alpine climates primarily because these regions have long been viewed to be temperature‐sensitive (e.g., Fridley, 2012; Harrington et al., 1989; Smith, 2013). As such, information is quite limited about the role of phenology in plant invasions under tropical or subtropical climates. Second, previous studies have mainly focused on spring‐flowering plants (Godoy et al., 2009; Smith, 2013; Wolkovich & Cleland, 2011; Wolkovich et al., 2013), and therefore, it is poorly known about the phenology of autumn‐flowering plants. Third, most studies have concentrated on one or two phenological phases but not on phenological sequences (but see Fernandes, Antunes, Correia, & Máguas, 2015). In fact, phenological sequences are more important than single phenological events in understanding the ecological roles of plant phenology (Fernandes et al., 2015; Post, Pedersen, Wilmers, & Forchhammer, 2008; Zhang, Post, & Shea, 2012).

Plant phenology is strongly driven by climate, and temperature is the most important driver of plant phenology (Lieth, 1974). Climate warming profoundly influences plant phenology (Cleland, Chiariello, Loarie, Mooney, & Field, 2006; Gerst, Rossington, & Mazer, 2017; Godoy et al., 2009; Hovenden, Wills, Schoor, Williams, & Newton, 2008; Novy et al., 2013), and invasive plants track climate warming more closely than native plants (Wolkovich et al., 2013). Aside from climate warming, atmospheric nitrogen (N) deposition is also among the key components of global change (Galloway et al., 2004). N deposition is likely to be serious in some regions invaded by exotic plants because N deposition and plant invasions occur at the same time in many cases. Cleland et al. (2006) found that N deposition delayed the flowering of grasses and slightly accelerated the flowering of forbs. In addition, plant community types affect plant phenology (Elzinga et al., 2007; Lieth, 1974), although this aspect has been overlooked (Dorji et al., 2013; Hülber, Bardy, & Dullinger, 2011). Similarly, invasion stages might influence the phenology of exotic plants. No studies to date have explicitly addressed the joint effects of climate warming, N deposition, and invasion stages on the phenology of plant invaders.

*Solidago canadensis* is a serious invader worldwide so that it has received increasing attention (Dong, Lu, Zhang, Chen, & Li, 2006). However, it should be noted that limited efforts have been paid to the impacts of global change on its phenology. Here, we did not consider its leaf phenology because the leaves of *S*. *canadensis* can emerge and die throughout a year in a subtropical climate. Reproduction is among the most important life history events (Harper, 2010). In general, reproductive phenology mainly includes the emergence of flower buds or inflorescence, flowering, and fruiting. These processes determine the quantity and quality of sexual propagules (Harper, 2010). Dieback is an important factor in determining growing season length (Li, Shao, Qiu, & Li, 2013). There has been little information about the dieback phenology of invasive plants and its ecological consequences (but see Li et al., 2009).

The purpose of this study was to understand the phenological sequences (i.e., first inflorescence buds, flowering, seed‐setting, and dieback) of autumn‐flowering invasive species under a subtropical climate in response to climate warming, N deposition, and invasion stages. To do so, we conducted a field experiment with *S*. *canadensis* and observed its four phenophases. Specifically, we focused on the following questions. How do experimental warming, N‐addition, and plant community types influence the phenological sequences of *S. canadensis*? Are the first inflorescence buds, flowering, seed‐setting, and dieback correlated with invasion success?

## 2 MATERIALS AND METHODS

### 2.1 Study species and experimental garden

*Solidago canadensis*, a worldwide invasive plant native to North America, has invaded several provinces in southeastern China and is expanding rapidly (Dong et al., 2006). Once established successfully, *S. canadensis* could form near or complete monocultures in invaded habitats, such as roadsides, abandoned fields, agricultural fields, and pastures. In its home range, the inflorescence of *S. canadensis* is first evident in late July or early August (Werner, Bradbury, & Gross, 1980). However, in its invaded range in China, it doesn’t start flowering until September. In our experiment, the seeds of *S. canadensis* were collected by hand from its 30 individuals in Ningbo, Zhejiang Province, a region invaded by it seriously, in 2011. These individuals had relatively similar genotypes because they were from the same plant community.

We also selected four native species (i.e., *Carex tristachya*,* Elymus dahuricus*,* Poa pratensis*, and *Solidago decurrens*) to assemble a native plant community. These four species were selected for the following reasons: they are common species in the invaded areas in China, and their congeners are also common in North America where *S. canadensis* is native.

**Figure 1:**
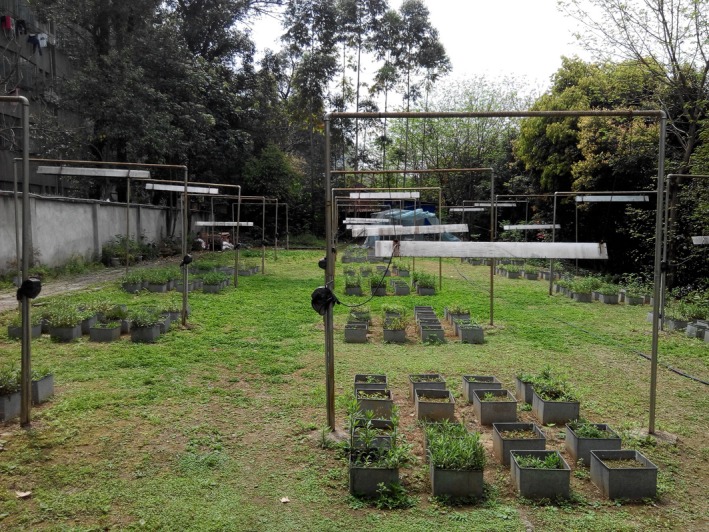
An image of the experimental garden and warming manipulation in 2012. Photograph credit: Y. Peng

To do this research, we established a common garden at Chengdu University of Technology (30.67°N, 104.06°E, and 512 m above sea level) in 2012. The garden was very flat and roughly occupied an area of 10 m × 20 m (Figure 1); it was characterized by mean annual precipitation of 918 mm and mean annual temperature of 16.2°C (1970–2000), and ferralsol. Climatically, this experimental garden was located in a subtropical region.

### 2.2 Experimental designs

We conducted a factorial field experiment at our common garden to answer the above‐mentioned questions. Three factors were warming, N‐addition, and community type, each with two levels. As suggested above, these factors are important drivers of plant phenology and their roles have been overlooked. Each experimental manipulation was replicated eight times, yielding 64 mesocosms (2 community types × 2 warming levels × 2 N levels × 8 replicates = 64 mesocosms). In April 2012, we collected local topsoil from several abandoned fields at Chengdu University of Technology, sifted all soils free from rocks, and mixed them with sand (1:1 volume) thoroughly. This procedure could allow soil properties (e.g., soil microbes and nutrients) to be relatively homogeneous. The homogenized soil was filled into 64 mesocosms (30 cm length × 30 cm width × 20 cm depth) with 16 10‐mm meshes at the bottom.

All plant assemblages were from seed. In early June 2012, 32 mesocosms were sown with *S. canadensis* seeds only to generate monocultures (analogous to late invasion stages), and the rest were sown with the seeds of *C. tristachya*,* E. dahuricus*,* P. pratensis*,* S. canadensis*, and *S. decurrens* to generate mixtures (analogous to early invasion stages). Seedlings were thinned gradually after seed germination and finally, only four seedlings per species occurred in each mesocosm. Accordingly, plant community types covered monocultures and mixtures, representing two typical communities invaded by *S. canadensis* in the field.

All the warming mesocosms were heated with a MSR‐2420 infrared radiator (Kalglo Electronics, Bethlehem, PA, USA) that was suspended 1.5–2.0 m above the soil surface during the experiment, roughly increasing air temperatures by 2°C (see below for details). This increase in air temperature was chosen according to the range projected by previous studies (IPCC, 2015; Meinshausen et al., 2009). For each unwarmed mesocosm, one “dummy” heater with the same shape and size was hanged as the infrared radiator to simulate the shading effect of the infrared radiator. Year‐round warming has been lasting since June 2012. We placed two data loggers (HOBO Pro v2) above community canopy under ambient and warming conditions, respectively. In other words, all data loggers were suspended at the same height. We recorded air temperatures at 5‐min intervals during the experiment.

Ammonium nitrate (NH_4_NO_3_) was selected as N source because it has been widely used in previous studies simulating N deposition. According to the previous predictions for future N deposition rates (Galloway et al., 2004), we used 4 g N m^−2^ year^−1^ as N‐addition rate. Specifically, N deposition was simulated by surface applications totaling 4 g N m^−2^ year^−1^; 1 g N m^−2^ was added in a wet pulse in March, April, May, and June each year. Meanwhile, the same amount of water was supplied to the mesocosms without N‐addition.

We weeded and supplied water to each mesocosm as necessary (e.g., the presence of obvious drought) during the course of the experiment. We monitored *S. canadensis* individuals per mesocosm and observed the onset of their first inflorescence buds, flowering, seed‐setting, and dieback from September to January of the subsequent year. The onset of inflorescence buds, flowering, seed‐setting, and dieback was defined as the presence of first inflorescence buds, open flowers, seed maturation, and dead shoots in each mesocosm, respectively. These phenophases were chosen for the following reasons: They are ecologically important and practically feasible, and different observers can identify them accurately. All phenological observations were implemented every 1–3 days, and these observations lasted from September 2014 to January 2017. From these observations, we separately determined the number of days from 1 January (i.e., day of year, DOY) for each phenological phase in each mesocosm. Note that the dieback of *S. canadensis* occurred in January of the subsequent calendar year so that the DOY for first dieback was calculated as the number of days from 1 January of the previous year. Therefore, the DOY values for dieback were greater than 365. Additionally, we surveyed ramet numbers and height in each mesocosm in late September, both of which can to some extent indicate invasion success.

### 2.3 Statistical analyses

We used a one‐way analysis of variance (ANOVA) to test whether mean air temperatures differed among three years or to test whether the phenological phases of *S. canadensis* monocultures grown under the ambient conditions differed among three years. Second, we used paired *t*‐test to test the warming effect on air temperatures. Third, a four‐way ANOVA was used to test the effects of experimental warming, N‐addition, community type, year, and their interactions on the phenology of *S. canadensis*. Finally, a model II regression was used to test the relationships between invasion success (i.e., ramet number and height) and each phenological phase. A model II regression was chosen as invasion success and phenophase were random variables and they were not mutually independent. All the ANOVAs and *t‐*test were carried out with SPSS 19.0 (SPSS Inc., Chicago, IL, USA), and a model II regression was performed using the package “smatr” (R Development Core Team, 2016).

## 3 RESULTS

**Figure 2:**
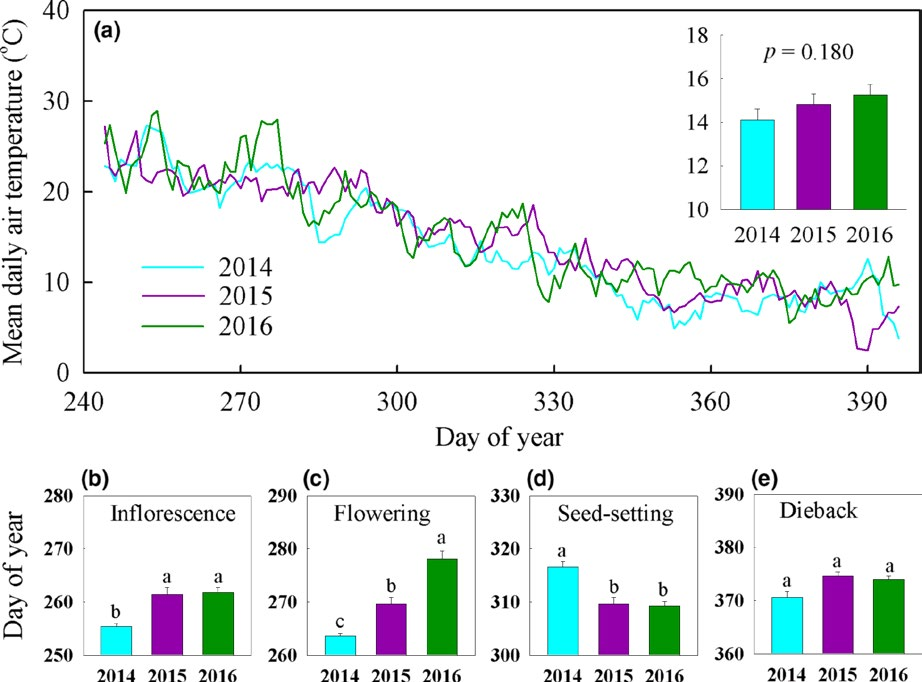
The air temperatures under ambient conditions and phenological sequences of *Solidago canadensis* therein. (a) The mean daily air temperatures under ambient conditions during the four phenological phases in 2014, 2015, and 2016; (b, c, d, and e) the onset of inflorescence buds, flowering, seed‐setting, and dieback of *S. canadensis* monocultures grown under ambient conditions. The inset in the main panel a represents the mean air temperatures over that time period. Data are presented as means + 1 *SE*. The bars with different letters in the main panels b, c, and d indicate a significant difference among years (*p *<* *0.05)

During the four phenophases of *S. canadensis*, the mean air temperatures under ambient conditions fluctuated from 14.1°C in 2014 to 15.3°C in 2016, but did not differ among three years (Figure 2a). Meanwhile, the precipitation was 143, 374, and 181 mm for 2014, 2015, and 2016 over that time period. For *S. canadensis* monocultures grown under the ambient conditions, the onset of first inflorescence buds (Figure 2b), flowering (Figure 2c), and seed‐setting (Figure 2d) shifted with years significantly. In contrast, the onset of first dieback retained unchanged with years (Figure 2e).

**Figure 3:**
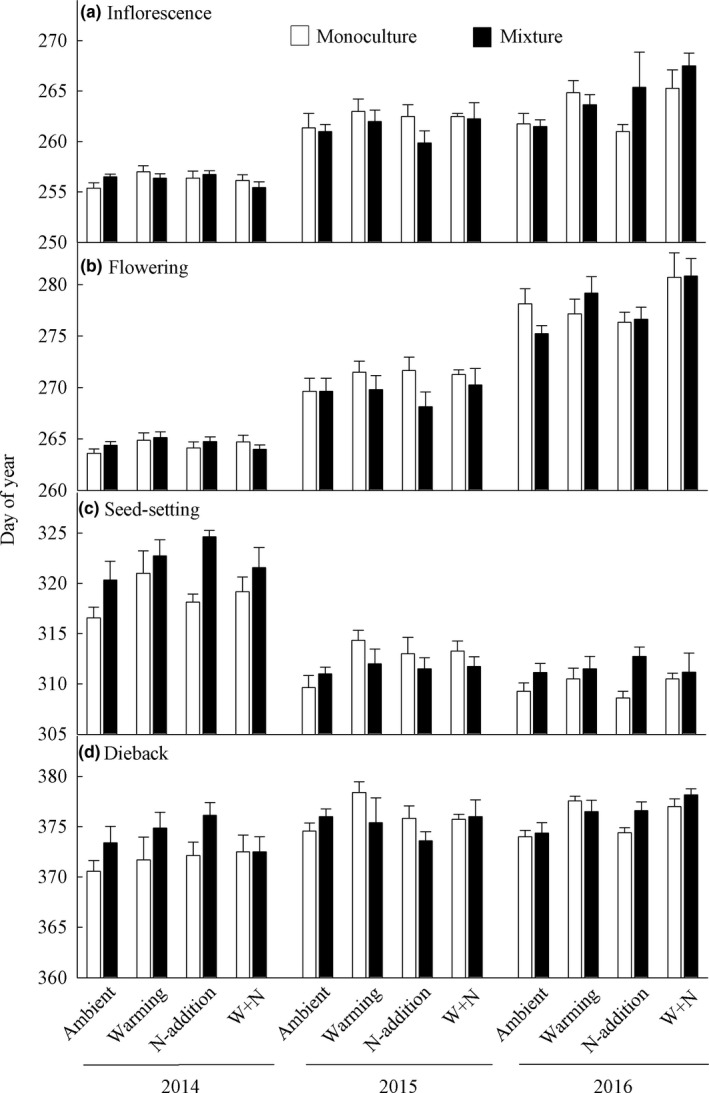
The phenology of *Solidago canadensis* individuals subject to eight manipulations in 2014, 2015, and 2016. (a) The onset of inflorescence buds, (b) the onset of flowering, (c) the onset of seed‐setting, and (d) the onset of dieback. W + N: warming and N‐addition at the same time; data are presented as means + 1 *SE*

Heating manipulations significantly increased air temperatures by 2.7°C in 2014, 1.9°C in 2015, and 1.7°C in 2016. Warming influenced the onset of first inflorescence buds, flowering, seed‐setting, and dieback; in contrast, N‐addition did not influence these four phenophases (Table 1). Plant community types influenced the onset of first seed‐setting rather than the other three phenophases (Table 1). Four phenophases varied with year significantly (Table 1; Figure 3a–d). Additionally, the interannual shifts in first seed‐setting and dieback were tightly dependent on plant community types (Table 1: interactive effects between year and community type; Figure 3c,d).

**Table 1:** The effects of experimental warming (W), N‐addition (N), plant community type (T), year (Y), and their interactions on the four phenological phases (i.e., first inflorescence bud date, first flowering date, first seed‐setting date, and first dieback date) of *Solidago canadensis*

Source	Inflorescence bud		Flowering		Seed‐setting		Dieback	
	*F*	*p*	*F*	*p*	*F*	*p*	*F*	*p*
Warming (W)	7.104	**0.009**	8.657	**0.004**	3.932	**0.049**	5.556	**0.020**
N‐addition (N)	1.113	0.293	0.791	0.375	0.847	0.359	0.278	0.599
Type (T)	0.029	0.865	0.980	0.324	7.794	**0.006**	2.180	0.142
Year (Y)	83.87	**<0.001**	286.4	**<0.001**	138.8	**<0.001**	14.95	**<0.001**
W × N	0.121	0.729	0.297	0.586	5.563	**0.020**	1.809	0.181
W × T	0.451	0.503	0.415	0.520	4.765	**0.031**	1.726	0.191
W × Y	2.901	0.058	2.475	0.088	0.332	0.718	2.342	0.100
N × T	0.832	0.363	0.214	0.645	0.270	0.604	0.073	0.787
N × Y	1.611	0.203	0.708	0.494	0.110	0.896	1.004	0.369
T × Y	1.615	0.203	1.207	0.302	6.015	**0.003**	3.492	**0.033**
W × N × T	0.153	0.696	0.084	0.772	0.021	0.885	0.228	0.634
W × N × Y	0.384	0.682	1.572	0.211	1.098	0.336	0.579	0.562
W × T × Y	0.529	0.591	1.043	0.355	0.116	0.891	0.061	0.941
N × T × Y	2.218	0.113	0.360	0.698	0.555	0.576	0.826	0.440
W × N × T × Y	0.323	0.725	1.810	0.167	0.813	0.446	2.355	0.099

Note

Values of *p *<* *0.05 are in bold.

N‐addition did not affect *S. canadensis* phenology, but it modified the warming effect on seed‐setting (Table 1: interactive effect between warming and N‐addition; Figure 3c). The first seed‐setting date of *S. canadensis* was 1.6 days earlier when it was grown alone than when it was grown with four natives (Table 1: effect of type; Figure 3c). The presence of natives also strongly modified the warming effect on seed‐setting (Table 1: interactive effect between warming and type). That is, warming delayed the first seed‐setting date of *S. canadensis* by 2.4 days when it was grown alone but did not influence this date when it was grown with natives (Figure 3c).

**Figure 4:**
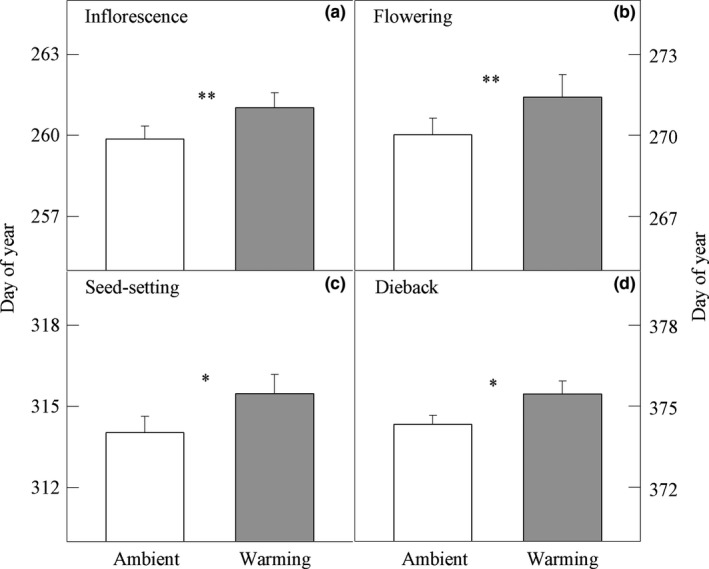
The phenology of *Solidago canadensis* individuals grown under two air temperature conditions across three years (i.e., 2014, 2015, and 2016). (a) The onset of inflorescence buds, (b) the onset of flowering, (c) the onset of seed‐setting, and (d) the onset of dieback. Data are presented as means + 1 *SE*. **: *p *<* *0.01; *: *p *<* *0.05

Across three years, experimental warming delayed the first inflorescence date by 1.3 days (Figure 4a), first flowering date by 1.4 days (Figure 4b), first seed‐setting date by 1.5 days (Figure 4c), and first dieback date by 1.2 days (Figure 4d) of *S. canadensis*. Taken together, 2°C climate warming might significantly delay these four phenophases of *S. canadensis*.

**Figure 5:**
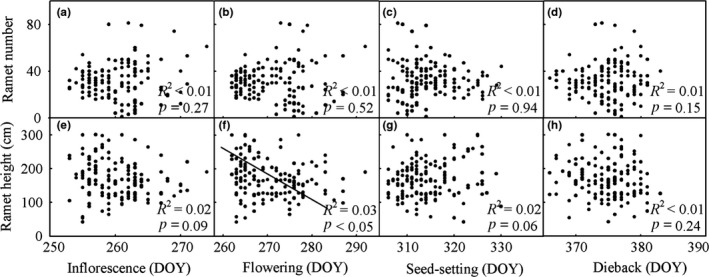
The relationships between invasion success (i.e., ramet number and height) and each of the four phenological phases (i.e., first inflorescence bud date, first flowering date, first seed‐setting date, and first dieback date) across three years (i.e., 2014, 2015, and 2016). Each data point represents the corresponding values of *Solidago canadensis* individuals grown in each mesocosm, which was subject to each of the eight experimental manipulations. A solid line represents a regression line at *p *<* *0.05. DOY: day of year

The total number of ramets per mesocosm was uncorrelated with the onset of first inflorescence buds (Figure 5a: *p *>* *0.05), first flowering (Figure 5b: *p *>* *0.05), first seed‐setting (Figure 5c: *p *>* *0.05), and first dieback (Figure 5d: *p *>* *0.05). The height of ramets was marginally or significantly correlated with the onset of first inflorescence buds (Figure 5e: *p *<* *0.1), first flowering (Figure 5f: *p *<* *0.05), and first seed‐setting (Figure 5g: *p *<* *0.1), but not first dieback (Figure 5h: *p *>* *0.05). Thus, reproductive phenology could indicate invasion success, as measured by ramet height.

## 4 DISCUSSION

Under ambient conditions, the first inflorescence buds, flowering, seed‐setting, and dieback of *S. canadensis* showed contrasting interannual patterns (Figure 2). These patterns suggest that plant phenological changes may be attributable to other factors than temperature and precipitation, and also support the traditional viewpoint that the factors determining plant phenology are diverse and complex (Lieth, 1974). For example, population size and photoperiod can alter plant phenology (Kouressy, Dingkuhn, Waksmann, & Heinemann, 2008; Tooke & Battey, 2010). Although soil microbes and genotypes can also influence plant phenology (Kouressy et al., 2008; Wagner et al., 2014), their role might be highly limited in this study due to the fact that experimental soils were homogenized and *S. canadensis* seeds were collected from the same plant community.

We found that experimental warming, N‐addition, and community types differentially influenced the phenology of *S. canadensis*, and they exhibited interactive effects on the first seed‐setting but not the other three phenophases (Figure 3). Our findings also highlight that *S. canadensis* might be sensitive to 2°C climate warming and insensitive to the current N deposition rates (i.e., 4 g N m^−2^ year^−1^).

Around 2°C warming caused the delayed onset of first inflorescence buds, flowering, seed‐setting, and dieback of the autumn‐flowering invader *S. canadensis* (Figure 4). Similar phenological delays due to climate warming have been detected in some spring‐flowering plants (Fitter & Fitter, 2002; Menzel et al., 2006). The delays in reproductive phases could be commonly attributed to the fact that warming delays the fulfillment of chilling requirements and thus leads to later onset of reproduction (Yu, Luedeling, & Xu, 2010 and references therein). Similarly, the delayed dieback due to warming could be ascribed to antecedent heat regimes, mainly temperatures of the preceding months (Badeck et al., 2004). The delayed dieback implies that warming could, to some extent, extend growing season length. Although climate warming influenced the performance of invasive and native plant species at the same time, it favored the colonization by invasive species over native species (Gillard, Thiebaut, Rossignol, Berardocco, & Deleu, 2017). Additionally, warming scenarios and magnitudes had differential effects on the performance of invasive and native plant species (Chen, Gao, Liao, & Peng, 2017).

Unlike our findings, Cleland et al. (2006) found that an addition of 7 g N m^−2^ year^−1^ significantly influenced the phenology of grasses and forbs. Therefore, the dose of N‐addition seems to matter in altering plant phenology. We also found that the delayed seed‐setting due to warming was evened out by N‐addition or the presence of native plants. More recently, Wan et al. (2017) reported that N availability regulated the effects of phosphorus on *S. canadensis* and Valliere, Irvine, Santiago, and Allen (2017) reported N availability modulated the effects of drought on native and nonnative species. Here we put forward a possibility for our finding. Warming enhanced the growth of *S. canadensis,* thereby speeding up soil N depletion; N‐addition could slow down this depletion directly, and the presence of native plants could slow down this depletion indirectly due to their slower growth (personal observations). Taken together, our findings highlight the importance of soil N availability in regulating the warming effects on some phenological events. Although an addition of 4 g N m^−2^ year^−1^ did not alter the phenology of *S. canadensis* directly, our findings might help us to understand how soil N availability influences the phenology of autumn‐flowering plants in the context of climate warming.

The most novel finding of our study was that the first seed‐setting date of *S. canadensis* was earlier in monocultures than in mixtures. We propose a hypothesis for this phenomenon. In mixtures, the overall growth of a plant community was relatively slower, depleting soil nutrients slowly; in contrast, the overall growth of a plant community was relatively faster in monocultures, depleting soil nutrients faster. Thus, the phenological shifts with community types might be closely linked to differential soil nutrient depletions. To date, information is limited about how biotic factors influence plant phenology (Elzinga et al., 2007; Wagner et al., 2014). This is especially true for invasive plants. In a general sense, mixed plant communities can roughly represent early invasion stages, and plant monocultures can represent late invasion stages. Our findings suggest that the onset of *S. canadensis* seed‐setting occurs later at early invasion stages than at late invasion stages. Although this shift did not occur in the first inflorescence buds, flowering, and dieback, this study provides evidence that some phenological phases of plant invaders may shift with invasion stages. In North America, *S. canadensis* inflorescence buds emerge in late July or early August (Werner et al., 1980); in China, their emergence occurs in mid‐ or late September (Dong et al., 2006). Such a shift between home versus invaded ranges appears to be related to invasiveness.

Reproductive phenology could, to some extent, indicate the invasion success of *S. canadensis*. When ramet height was chosen as a proxy of invasion success, it was associated with the onset of first inflorescence buds, flowering, and seed‐setting (Figure 5). In other words, reproductive phenology contributed to invasion success. In contrast, there were no relationships between invasion success and reproductive phenophases when ramet numbers were used as a proxy of invasion success (Figure 5). In the field, *S. canadensis* is a typical clonal plant, has a strong capacity to yield offspring asexually, and ramet numbers are primarily determined by clonal growth (Dong et al., 2006; Werner et al., 1980). This clonality confers competitive advantages to *S. canadensis* and thus contributes greatly to its invasion success (Dong et al., 2006). This viewpoint is consistent with our recent field observations (Wei‐Ming He, Li‐Jia Dong, & Hong‐Wei Yu, unpublished data). Meanwhile, this clonality might help to explain why ramet numbers were not correlated with reproductive phenology. Note that the nature of ramet growth is vegetative growth (i.e., clonal growth), which is tightly linked to biomass accumulation over the growth period. In this sense, the production of ramets may be closely associated with growth season length. As discussed above, warming could expand growth season and thus might enhance ramet production. Importantly, *S. canadensis* can yield new leaves throughout a year, which might be beneficial for its individuals to accumulate biomass. Our data also highlight that it is important to distinguish sexual and asexual reproduction when addressing the role of phenological sequences in driving plant invasions.

Plant phenology is a key aspect of life history strategies to cope with changing environments (Chuine & Régnière, 2017). This study advances our understanding of the effects of climate warming, N deposition, and invasion stages on the phenological sequences of autumn‐flowering invaders in a subtropical climate. Briefly, symmetric 2°C climate warming might delay the phenology of autumn‐flowering invaders. The current N deposition rates might not influence the phenology of autumn‐flowering invaders. Among the four phenophases observed, seed‐setting was most sensitive to manipulations. Thus, this phenophase appears to be a good indicator of global change. Importantly, our findings provide primary evidence that phenological cues may have payoffs for plant invaders. Invasion stages might influence the phenology of plant invaders, and the reproductive phenology of invaders might be linked to their invasion success.

## AUTHOR CONTRIBUTIONS

W.M.H., P.H.P., and J.J.L. designed the experiment. Y.P., J.X.Y., and X.H.Z. performed the experiment and collected the data. Y.P., J.X.Y., and W.M.H. analyzed the data. W.M.H., Y.P., and J.X.Y. wrote the manuscript. All authors contributed critically to the drafts and gave final approval for publication.
